# High-frequency measurement of concentration in an isothermal methane–air gas mixture using spontaneous Raman spectroscopy

**DOI:** 10.1038/s41598-023-37649-1

**Published:** 2023-08-01

**Authors:** Jocelino Rodrigues, Lee Weller, Francesca De Domenico, Simone Hochgreb

**Affiliations:** 1 Department of Engineering, University of Cambridge, Cambridge, CB2 1PZ UK; 2 Aerospace Engineering, Delft University of Technology, Kluyverweg 1, 2629 HS Delft, The Netherlands

**Keywords:** Raman spectroscopy, Engineering, Raman spectroscopy, Characterization and analytical techniques, Design, synthesis and processing

## Abstract

A high-frequency (1.5 kHz) spontaneous Raman spectroscopy measurement technique is developed and applied to measure external fluctuations generated in the local concentration of an isothermal binary gas mixture of methane and air. Raman excitation is provided by a high-frequency laser at 527 nm in dual-pulsed mode. The Stokes Raman signal is collected using an EMCCD camera coupled to a high-frequency intensifier as a shutter. The emitted signal is collected over the 596–627 nm wavelength range, which allows for the simultaneous tracking of methane and nitrogen Stokes *Q*-branch mode signals. Calibration curves are initially obtained for each species (${\text{CH}}_{4}$ and ${\mathrm{N}}_{2}$) based on steady-state concentrations, and further corrected during use to detect local unsteady mixture fluctuations at gas pulsation frequencies up to 250 Hz. The main novelty is the demonstration of Raman spectroscopy for the simultaneous multispecies measurement of unsteady concentrations of gas-phase methane and air mixtures using a laser beam with a high-repetition rate, low energy per pulse, combined with a high-frequency intensifier and a single camera.

## Introduction

The in situ measurement of unsteady local concentrations of species in gases is a common need across various problems in reacting and non-reacting flows—for example, to determine catalytic reactivity^1^, to provide data for species mixing models^2^, or to study thermoacoustic instabilities^3,4^. However, few options are available for high-frequency (kHz) measurements. Tunable Diode Laser Absorption Spectroscopy (TDLAS)^4–11^ has been used for high-frequency line of sight measurements. Fluorescence can be used for fast local measurements, if species have accessible transitions with sufficient line strength, such as the case with NO, OH, and specific species which can be accessed with continuous wave^12^ or high-frequency pulsed lasers^13^. Developments in high-frequency burst mode lasers have enabled single-shot and multispecies Raman spectroscopy of species^14–17^, single-shot measurements of species using grating spectroscopy^18^, as well as Coherent Anti-Stokes Raman (CARS) measurements of species^19^. However, these experiments require highly specialized facilities with a very complex and expensive setup. Moreover, pulse burst lasers are limited to providing high-repetition rate pulses only for a short amount of time, followed by a long time to cool down. Rayleigh and filtered Rayleigh scatter measurements are bulk measurements, and therefore not species-specific. Further, Rayleigh scatter (but less so filtered Rayleigh scatter) are less suitable for systems in enclosures, where scattered light at the same wavelength as the pump would be collected (e.g. from walls, particles, etc.), and difficult to filter out in any sensible way. Thus, for any applications where identifying the species is key, or experiments in enclosures, such measurements are much less appropriate, and have to be combined with Raman scattering^20^.

Spontaneous Raman scattering measurements^21–23^ are based on the frequency shift of the emitted photon relative to the incident one, as it relates to the rovibrational modes of the probed species^24,25^. In particular, the Raman shift $\Delta \tilde{\nu }$ is reported in wavenumbers (units of cm$^{-1}$) and is calculated using $\Delta \tilde{\nu } = \left( 1/\lambda _{p} - 1/\lambda _{S} \right) \times 10^7$, where $\lambda _{p}$ is the pump (or excitation) wavelength and $\lambda _{S}$ is the spectral (or emitted) wavelength in nm. High-speed pulsed lasers have not often been considered for high-frequency Raman spectroscopy measurements in gases since the Raman optical cross-sections are low, and laser powers of typical pulsed lasers insufficient. To overcome low laser energies, Raman signals can be amplified using multi-pass cavity techniques, however this limits time resolution^26^. Recent technological advances have introduced high-frequency lasers providing increased energies per pulse and adequate time resolution^1,27–29^. However, there is a trade-off between the repetition rate (i.e. frequency resolution of the measurement) and the energy delivered per pulse. This translates to a trade-off between the signal-to-noise ratio and the temporal resolution which can be obtained with the system. In this work, a high-frequency (1.5 kHz), low energy, non-burst spontaneous Raman spectroscopy system is developed for the purpose of measuring and characterizing the local species concentrations of a non-reacting isothermal binary gas mixture (methane–air).

The original motivation for the present work is the study of the indirect noise generated by the acceleration of compositional inhomogeneties (or compositional waves)^30–32^. The acceleration of compositional waves has been shown to be responsible for the generation of sound (i.e. indirect noise), which can contribute to the overall noise generated in a combustion chamber^33,34^. Indirect noise transfer functions are used to predict how much noise a nozzle will produce for a given compositional fluctuation^32,34^ and are crucial to the understanding of an engine’s thermoacoustic stability^35^. Model thermoacoustic systems have been developed to study convected compositional inhomogeneties in simplified flows with well-controlled conditions^31,32,36^. In order to quantify indirect noise transfer functions, the convecting compositional fluctuations must be accurately measured at a wide range of frequencies^37,38^. While the composition-to-sound conversion phenomenon goes beyond the scope of this paper, this work focuses on the application of high-speed Raman spectroscopy to time-resolve the amplitude of convecting compositional inhomogeneities.Specifically, we propose a relatively modest and simple single-camera setup for applications where simultaneous multispecies local measurements on a millisecond time scale are sufficient. Table 1 compares a select number of Raman spectroscopy experiments in literature for multispecies measurements to demonstrate the differences and similarities between past literature and this present work.

This paper is organized as follows. We first present the experimental spontaneous Raman scattering setup and the test section. The data processing and calibration procedures are then described using steady-state experiments, followed by the measurement of unsteady concentration fluctuations.

**Table 1:** Comparison of select Raman spectroscopy multispecies measurement experiments in literature presented in chronological order. From left to right—publication details: first author and peer-reviewed publication ($^{\dagger }$NASA technical memorandum, $^{\dagger \dagger }$conference manuscript); Raman diagnostics setup: burst mode operation, repetition rate, approx. energy per shot, multi-pass cavity technique used, single-camera setup used for multispecies measurement ($^{*}$1 camera per species); measurement details: concentration, time series data presented, SNR value(s) quantified and quoted in the manuscript: Y = yes; N = No; Bold = positive metric; Italics = negative metric.

Publication		Diagnostics setup					Measurements		
First author	Peer reviewed	Burst mode	$f_{\mathrm{rep}}$ (Hz)	E/shot (mJ)	Multi-pass cavity	Single camera	Concentration	Time series	SNR value(s) quoted
Kojima^3^	$N ^{\dagger }$	**N**	*1000*	*30*	**N**	**Y**	*N*	**Y**	*N*
Gabet^15^	$N^{\dagger \dagger }$	*Y*	**10,000**	**400**	**N**	**Y**	**Y**	**Y**	**Y**
Jiang^16^	**Y**	*Y*	**10,000**	**750**	**N**	**Y**	*N*	**Y**	*N*
Yang^17^	**Y**	*Y*	*5000*	**450**	**N**	$N^{*}$	*N*	**Y**	*N*
Guiberti^27^	**Y**	**N**	*10*	**1100**	**N**	$N ^{*}$	*N*	*N*	*N*
Tang^28^	**Y**	**N**	*10*	**1000**	**N**	**Y**	*N*	*N*	*N*
Tang^29^	**Y**	**N**	*10*	**1000**	**N**	**Y**	**Y**	*N*	**Y**
Kim^26^	**Y**	**N**	**10,000**	*3*	*Y*	**Y**	*N*	*N*	**Y**
Present work	**Y**	**N**	**1500**	**38**	**N**	**Y**	**Y**	**Y**	**Y**

## Experimental setup

### Raman spectroscopy system

**Figure 1:**
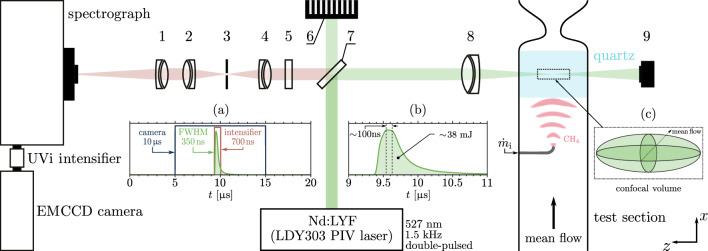
Schematic of the experimental setup. The main components of the Raman system are: (1) achromatic doublet lens (Ø = 25.4 mm, *f* = 100 mm), (2) achromatic doublet lens (Ø = 25.4 mm, *f* = 50 mm), (3) mounted pinhole (Ø = 25.4 mm, 25 $\upmu$m), (4) achromatic doublet lens (Ø = 25.4 mm, *f* = 50 mm), (5) 532 nm single-notch filter (Ø = 25.4 mm), (6) beam trap, (7) long-pass 532 nm dichroic beamsplitter (Ø = 25.4 mm), (8) achromatic doublet lens (Ø = 50.8 mm, *f* = 100 mm), (9) photodiode (PD) detector. Also included: (**a**) timings of the camera-intensifier-laser system, (**b**) representative energy time history for a combined two-beam pulsed delivery, and (**c**) confocal volume schematic (oblate ellipsoid). Dimensions not to scale.

The optical setup is described in Fig. 1. A Litron LDY303 dual head laser system was used to generate the excitation radiation at 527 ± 0.01 nm (spectral half-width of approximately 6 GHz or $\delta \tilde{\nu }_p$ = 0.2 cm$^{-1}$)^39^. The laser system was used in dual-pulsed mode whereby two pulses were combined (with an estimated 100 ns time separation) to get approximately 38 mJ in the combined 350 ns FWHM pulse at a rep rate of 1.5 kHz (Fig. 1a,b). The laser beams were guided by various high reflectivity 532 nm mirrors onto a long-pass 532 nm dichroic beamsplitter (Thorlabs LPD02-532RU-25). Most of the 527 nm light was reflected by the beamsplitter towards an achromatic doublet lens (Ø = 50.8 mm, *f* = 100 mm) (Thorlabs AC254-100-A-ML) which focused the light into the quartz test section. The collection volume is estimated from the delivery and collection geometry as a shape of length of approximately 1−5 mm along the *z*-axis (cross-stream direction, as defined in bottom right-hand corner of Fig. 1), with a beam diameter of 5 ± 2 mm (assuming a confocal sample volume – see Fig. 1c)^36^, for a total volume of 0.5−12.0 $\times$ 10$^{-4}$ mm$^3$. A photodiode detector (Thorlabs PDA10A2), positioned on the opposing side of the measurement point captured the shape and relative amplitude of the laser pulses in time. A backward-scattering configuration was used, made possible by the use of the dichroic beamsplitter, which reflected the excitation light, while efficiently passing the longer back-scattered Raman-shifted (Stokes) wavelengths.

A 532 nm single-notch filter (Thorlabs NF01-532U-25) was used to isolate the lower energy Raman-scattered light. The collimated light was then focused by an achromatic doublet lens (Ø = 25.4 mm, *f* = 50 mm) (Thorlabs AC254-050-A-ML) through a 25 $\upmu$m pinhole (Thorlabs P25C) and was then re-collimated using another achromatic doublet lens (Ø = 25.4 mm, *f* = 50 mm) (Thorlabs AC254-050-A-ML). The Raman-scattered photons were then focused by an achromatic doublet lens (Ø = 25.4 mm, *f* = 100 mm) (Thorlabs AC508-100-A-ML) onto the aperture of an Andor Shamrock SR-303i spectrograph which was used to collect the Raman-scattered light at wavelengths ranging from 596 to 627 nm (Raman shifts of 2197 $\le$ $\Delta \tilde{\nu }$ $\le$ 3008 cm$^{-1}$) with an average wavenumber resolution of approximately 13 cm$^{-1}$. The spectrograph had a side input slit size of 200 $\upmu$m and an F/4 aperture. The diffraction grating inside the spectrograph had 1200 lines/mm and was blazed at 500 nm. An Invisible Vision UVi camera intensifier (1850-10-S20) was connected to the output of the spectrograph with an exposure of 700 ns and a delay of 4.4 $\upmu$s, as shown in Fig. 1a. This exposure interval was chosen as a trade-off between a long enough window to collect and amplify the signal, while minimizing noise from excited fluorescence arising from the optics and the quartz tube test section (more details in section “Test section”). The intensifier gain *G* could be set between a nominal 1% to 100%, defined as a percentage between the minimum and a maximum voltage applied to the intensifier's microchannel plates (chevron pair)^40^. These correlate to roughly 970 V (for *G* = 1%) and 1705 V (for *G* = 100%) across both plates (approximately evenly split), with a linear increase in voltage between those two limit values (i.e. $G~[V]~\simeq ~7.42~G~[\%] + 963$)^40^. The intensifier multiplication was set to the default value EM = 15 (unitless).

The intensifier was coupled to an Andor iXon Ultra-888 back-illuminated EMCCD camera which was Peltier-cooled to -60$^{\circ }$C before experiments were run. The camera’s exposure interval was set to 10 $\upmu$s, as shown in Fig. 1a. Nevertheless, the camera was only exposed to light during the opening interval of the intensifier gate (700 ns). The camera was run in kinetic acquisition mode with an external trigger from the laser. The output amplifier of the camera was set to electron multiplying, but the electron multiplication gain was not used. The chip was set to frame transfer (optically centered ROI), with crop mode (1024(W) $\times$ 32(H)) and a binning of 16 $\times$ 16, which allowed for a frequency of detection of 1.5 kHz. A background reference image was taken before collecting each signal. Preliminary experiments showed that drift in the camera background signal limited the acquisition window to a maximum of 1500 shots (or 1 second) per test case^36^. A 4-channel Teledyne LeCroy 6104A High Definition oscilloscope was used to record the trigger signals to the intensifier, the camera, as well as the output signal from the photodiode (PD) detector. Each shot (or segment) recorded 2.5 kS (kilo samples) at a rate of 8 ns per sample, capturing data over 20 $\upmu$s per shot.

### Test section

The test section where spontaneous Raman spectroscopy measurements were made is identified in Fig. 1. This is a model setup designed primarily for the study of the effect of compositional disturbances on pressure fluctuations associated with the passage of the distinct compositional fluctuations through a nozzle; similar versions of the setup have been used in previous work^31,32,41^. Compositional disturbances in the form of suddenly injected pulse trains of methane are generated into a mean flow of air, and then advected through the tube. As part of the present work, two experimental campaigns were performed at isothermal conditions: steady-state and unsteady. The mean flow for both campaigns was delivered to a 520 mm long duct (test section) with a constant area (40 mm ID), as shown in Fig. 1.

**Figure 2:**
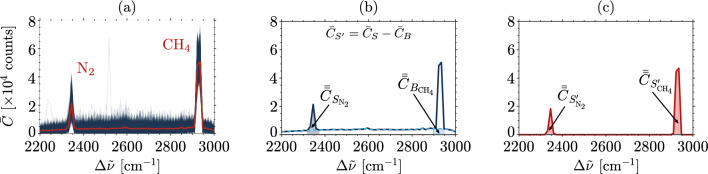
Methane–air mixture $Y_{{\mathrm {CH}}_{4}}$ = 20% at atmospheric pressure ($M_{{\mathrm {CH}}_{4}}$ = 13.0 mol m$^{-3}$, $M_{{\mathrm {N}}_2}$ = 22.4 mol m$^{-3}$) averaged over $N_s$ = 1450 shots with intensifier gain *G* = 55%: (**a**) energy-normalized counts *C *() and averaged counts $\bar{C}$ (); (**b**) averaged counts for the full Stokes signal ($\bar{C}_S$ ) and for the fitted background signal ($\bar{C}_B$ ) where it is assumed that $\bar{C}_{S'}$ = $\bar{C}_{S}$ − $\bar{C}_{B}$; (**c**) background-subtracted averaged counts ($\bar{C}_{S^\prime }$ ) and integrated counts ($\bar{\bar{C}}_{S^{\prime }_{\mathrm {N_2}}}$ and $\bar{\bar{C}}_{S^{\prime }_{\mathrm {CH_4}}}$).

For the steady-state experiments, the mean flow was composed of either (a) a fully premixed methane–air flow with a set composition, (b) a pure air flow, or (c) a pure methane flow (depending on the test case). Methane was selected for the experimental campaigns as it has a large differential Raman scattering cross-section d$\sigma /$d$\Omega$^42^ (variable which is proportional to the laser-induced Stokes photons and counts, as shown in Eq. (1)). In addition to this, the Stokes Raman shift $\Delta \tilde{\nu }_S$ of methane is near that of nitrogen, which allows tracking of both molecules concurrently (Fig. 2) using the setup described in section “Raman spectroscopy system”.

The air mass flow rate was controlled using an Alicat MCR2000 mass flow controller (max. flow rate: 2000 slpm, accuracy: ± 1%). The flow rate of methane (BOC, impurities: ${\mathrm {O}}_{2}$ = 25 ppmv; ${\mathrm {N}}_{2}$ = 1000 ppmv; ${\mathrm {H}}_{2}{\mathrm{O}}$ = 10 ppmv; ${\mathrm {C}}_{2}{\mathrm{H}}_{4}$ and other HCs = 3000 ppmv) was controlled by adjusting the pressure downstream of the methane gas tank and by subsequently monitoring the flow in the line using an Alicat M100 mass flow meter (max. flow rate: 100 slpm, accuracy: ± 1%FS). The two gas lines (air and methane) were connected by a y-piece. A flexible polyurethane tube (SMC TU1208, 8 mm ID) was used to deliver the mixture to a 2.1 m long piston (modified ISO 15552 pneumatic cylinder) where the gases were allowed to fully mix before entering the main test section. For the pure air flow experiments, test cases were run with and without a convergent-divergent nozzle termination to assess the effects of pressure (slightly above atmospheric) on the signal. For the steady experiments, no unsteady injection of methane took place.

For the unsteady experiments, the mean flow was composed of air and, for all test cases, the air mass flow rate was set to $\bar{\dot{m}}$ = 6 g s$^{-1}$ (mean flow bulk velocity of $\bar{u}$ = 3.7 m/s in the test section). Fluctuations in concentration were generated via the pulse train injection of methane (Fig. 1) using a high-speed SMC SX10 series 80 W two-port solenoid valve. The exact model (SX11F-AH; SX11F-EH; SX11F-JH) was case dependent in order to try to meet the specified injected mass fraction and minimize the valve response time for the particular case (Table 2). The valve was controlled by square pulse trigger signal from the laser system. The injected gas entered the duct at the centreline of the main test section via a 90$^\circ$ L-bend pipe with an inner diameter of 3.4 mm. The mass flow rate of injected gas $\dot{m}_{\mathrm{i}}$ was monitored using an Alicat M100 mass flow meter (max. flow rate: 100 slpm, accuracy: ± 1%). Since the injected pulse widths were short ($t_p$ $\le$ 20 ms) and the mass flow meter has a low sampling frequency (approximately 35 Hz), the injected flow rate cannot be accurately time-resolved by the mass flow meter. The main test section was connected to a circular quartz section with 45 mm length of optically accessible length. For all unsteady test cases, the test section was terminated by a convergent-divergent nozzle (as shown in Fig. 1). The injection plane was approximately 62 mm upstream of the probe volume and 107 mm upstream of the nozzle.

## Steady-state experiments

This section presents details on the data processing, calibration procedure, and results for steady isothermal concentration measurements of methane–air mixtures for different conditions.

### Data processing

The rate of laser-induced Stokes photon emission $\gamma _{S}$ (photons s$^{-1}$) can be described by^43–45^:

1 $$\begin{aligned} \gamma _{S}=\bigg (\frac{48({\mathrm{NA}})^{4}}{111.84 \pi ^{1/2} {n}^{4}}\bigg )\bigg (\frac{P_{\mathrm{in}}}{h\delta \tilde{\nu }_{S}\delta \tilde{\nu }_{p}}\bigg ) \bigg (\frac{{\nu }_{p}}{{\nu }_{S}}\bigg )^{3} \bigg ({c N_A \frac{{\mathrm{d}}\sigma }{{\mathrm{d}}\Omega }}\bigg )M, \end{aligned}$$

where ${\mathrm{NA}}$ is the numerical aperture of a lens (${\mathrm{NA}} = d/2f$), *c* is the speed of light, *n* is the index of refraction, $N_{A}$ is Avogadro’s constant, *M* is the molar concentration, $\nu _{S/p}$ is the Stokes/pump frequency, $\delta \tilde{\nu }_{S/p}$ is the Stokes/pump mode half-width at half maximum, ${\mathrm{d}}\sigma /{\mathrm{d}}\Omega$ is the differential Raman scattering cross-section (with a $\lambda _{p}^{-4}$ dependence^43,46,47^), $P_{\mathrm{in}}$ is the pump input power.

The total signal collected by the camera across the spectrometer $C_{\mathrm{raw}}$ is proportional to the rate of photons emitted, integrated across the fixed time interval $\Delta t$, where *k* is a constant representing the ratio of signal count rate to total photon rate. The factor *k* includes the transfer function of all optical elements (spectrometer, intensifier, and optics) at the Stokes and pump wavelengths:

2 $$\begin{aligned} C_{\mathrm{raw}} = k \int _0^{\Delta t} \gamma _S \; dt. \end{aligned}$$

The total counts are normalized by the corresponding measured relative laser pulse energy, as monitored by the integrated signal of the PD detector $\mathscr {V}$. The average measured laser pulse shape in time is shown in Fig. 1a. The averaged signal $\bar{C}$ for the total number of shots $N_s$ is calculated as an ensemble average over $N_s$:

3 $$\begin{aligned} \bar{C} = \frac{1}{N_s} \sum _{i=1}^{N_s} C_{i}, \end{aligned}$$

where

4 $$\begin{aligned} C_{i} = \frac{\bar{\mathscr {V}}}{\mathscr {V}_i} C_{{\mathrm{raw}},i} \qquad \text {for} \qquad 1 \le i \le N_s, \end{aligned}$$

where the mean integral $\bar{\mathscr {V}}$ of the PD signal *V* is:

5 $$\begin{aligned} \bar{\mathscr {V}} = \frac{1}{N_s} \sum _{i=1}^{N_s} \mathscr {V}_i = \frac{1}{N_s} \sum _{i=1}^{N_s} \int _{t_0}^{t_f} V_{i}(t) \,dt, \end{aligned}$$

where $t_0$ and $t_f$ are the start and finish times for each shot, respectively, and the integrand $V_i(t)$ is the PD voltage signal, which is assumed to be proportional to laser energy per unit time. The ratio of the standard deviation and the mean of the photodiode signal measurements was between 2.5–5%.

Figure 2a shows the energy-normalized counts *C* and averaged counts $\bar{C}$ for a calibration test case. Raman-scattered wavelengths for both nitrogen and methane are captured simultaneously by the setup (2200 < $\Delta \tilde{\nu }$ < 3000), which is used for calibration of the unsteady concentration fluctuations, by checking that the sums of molar fractions add up to unity (see section “Phase-averaged signal”). The background signal $\bar{C}_{B}$ is estimated as shown in Fig. 2b. This broadband background signal is caused by CCD camera noise sources (e.g. dark current, photon shot noise and readout noise^48^) and intensified luminescence (fluorescence and phosphorescence) from the cylindrical quartz tube (SiO_2_)^49–51^. The background subtraction is done by fitting a linear function to the $\bar{C}$ signal^52^. A background-subtracted signal $\bar{C}_{S'}$ is obtained by subtracting $\bar{C}_{B}$ from the total signal $\bar{C}_{S}$, such that $\bar{C}_{S'}$ = $\bar{C}_{S}$ − $\bar{C}_{B}$. This procedure is demonstrated in Fig. 2b,c. The profiles are integrated in wavenumber space to obtain the total integrated counts per shot $\bar{\bar{C}}$. For the background-subtracted signal and for the background signal, respectively:

6a-b $$\begin{aligned} \bar{\bar{C}}_{S^{\prime }} = \int _{\Delta \tilde{\nu }_{1}}^{\Delta \tilde{\nu }_{2}} \bar{C}_{S^{\prime }} \,d\tilde{\nu }, \qquad \qquad \bar{\bar{C}}_{B} = \int _{\Delta \tilde{\nu }_{1}}^{\Delta \tilde{\nu }_{2}} \bar{C}_{B} \,d\tilde{\nu }, \end{aligned}$$

where $\Delta \tilde{\nu }_1$ and $\Delta \tilde{\nu }_2$ are the bounding wavenumbers for the respective species peaks. For the experiments presented here: $\Delta \tilde{\nu }_{1} ({\mathrm{N}}_2)$ = 2319 ${\mathrm {cm}}^{-1}$ and $\Delta \tilde{\nu }_{2} ({\mathrm{N}}_2)$ = 2372 ${\mathrm {cm}}^{-1}$ (mode width of 53 cm$^{-1}$); $\Delta \tilde{\nu }_{1} ({\mathrm{CH}}_4)$ = 2898 ${\mathrm {cm}}^{-1}$ and $\Delta \tilde{\nu }_{2} ({\mathrm{CH}}_4)$ = 2947 ${\mathrm {cm}}^{-1}$ (mode width of 49 cm$^{-1}$). The values are in agreement with published locations of Stokes signals^42,53–58^, within the limitations of the wavenumber resolution of the setup. Variability of the mode half-widths $\delta \tilde{\nu }$ and Raman shifts $\Delta \tilde{\nu }$ of both species due to changes in mean absolute pressure^59–61^, temperature^62^ or concentrations^63,64^ are expected to be within the spectral resolution of the system.

For each test condition, the uncertainty associated with the measurement of the integrated counts $\bar{\bar{C}}_{S'}$ is computed by taking the standard deviation $\sigma _{\bar{\bar{C}}_{S'}}$ of the single-shot integrated counts (an example of shot-to-shot variability is shown in Fig. 2a). This value accounts for all the measurable fluctuations in the system.

### Calibration measurements

In this section, the high-speed Raman spectroscopy setup is calibrated using steady-state test cases (i.e. fully mixed) at isothermal conditions. This was done by varying the methane mass fraction $Y_{{\mathrm{CH}}_4}$ (0–100%), intensifier gain *G* (35–70%), and mean absolute pressure $\bar{p}$ (101–153 kPa) for a total of 144 test cases (where $N_s$ = 1450 shots were recorded for each test case). A mean flow temperature $\bar{T}$ of 293.15 K is assumed to be constant (i.e. there is no heat source or heat sink). The expected composition of the air is 78.1% (${\mathrm {N}}_2$), 21.0% (${\mathrm{ O}}_2$), 0.9% (other).

**Figure 3:**
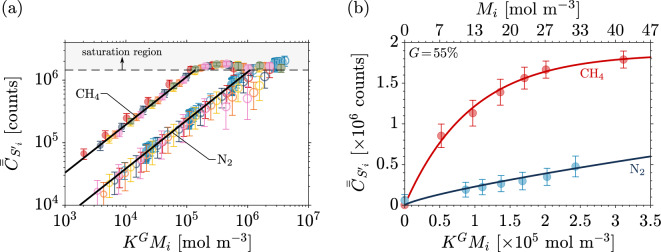
Steady-state signal as a function of scaled molar concentration for: (**a**) nitrogen (empty circles) and methane (filled circles) for 144 test cases at different mean absolute pressures $\bar{p}$ (101–153 kPa), methane mass fractions $Y_{{\mathrm{CH}}_4}$ ( 0%,  10%,  20%,  30%,  40%,  50%,  100%), and intensifier gains *G* (35–70%)—circle sizes are proportional to *G*. Error bars show the signal standard deviation (± 1$\sigma$) for each test case for a series of $N_s$ = 1450 shots; (**b**) nitrogen () and methane () at atmospheric pressure ($\bar{p}$ = 101,325 Pa) and *G* = 55% (gain used in the unsteady experiments presented in section “Unsteady experiments”).

For each test case and species, the integrated counts $\bar{\bar{C}}_{S'}$ are plotted in Fig. 3a as a function of molar concentration *M* of species *i* scaled by an intensifier factor $K^G$ = log$_{10}$(EM)$^G$. The power-law dependence between the signal and intensifier gain arises from the microchannel plate electron current behavior, which doubles with every 50 V applied^40^. The electron multiplication factor EM = 15 is constant for all test cases. Therefore, *K* = log$_{10}$(EM) = 1.176 is also a constant for all test cases. The error bars show the standard deviation (± 1$\sigma$) for each test case. The shot-to-shot signal variation is visibly larger for nitrogen than for methane. As such, the maximum measured counts-based signal-to-noise ratio ${\mathrm{SNR}}$ = $\bar{\bar{C}}_{S'}/\sigma _{\bar{\bar{C}}_{S'}}$ in the non-saturated region is 4.4 for nitrogen and 10.6 for methane (coefficients of variation of 22.5% and 9.5%, respectively)—see Appendix A.2 . The calibration measurements in the non-saturated region presented in Fig. 3a can be fit by a power-law relationship:

7 $$\begin{aligned} \bar{\bar{C}}_{S'_i} = \beta _i (K^{G} M_i)^{\eta }, \end{aligned}$$

where $\eta$ = 0.77 is a constant and $\beta _i$ is specific to each species ($\beta _{{\mathrm {CH}}_4}$= 165, $\beta _{{\mathrm {N}}_{{2}}}$= 31.4). Additional details on how the region of saturation was identified and the curve fits were calculated are available in Appendix A.1.

In the unsteady experiments presented in section “Unsteady experiments”, an intensifier gain of *G* = 55% was used. The calibration curves for nitrogen and methane measurements for this gain are shown in Fig. 3b. The nitrogen signal measurements for *G* = 55% shown in Fig. 3b follows the same fit as the one presented in Fig. 3a and described by Eq. 7. In the case of methane, however, a power fit is not sufficiently accurate, and instead a saturation curve (exponential decay increasing form) is used:

8 $$\begin{aligned} \bar{\bar{C}}_{S'_{{\mathrm{CH}}_4}}=\vartheta \big (1-e^{-\Upsilon K^G M_{\mathrm{CH}_4}}\big ), \end{aligned}$$

where $\vartheta$ = 1.86 $\times$ $10^6$ is the limiting (or asymptotic) value and $\Upsilon$ = 1.06 $\times$10$^{-5}$ is the growth rate. The average error on the steady-state curves is 7% across the 7 data points (minimum error of 0% and maximum error of 14%). The error is a function of concentration as it directly relates to how much the fit deviates from each experimental data point.

## Unsteady experiments

### Test cases

Many unsteady flow applications have concentration fluctuations on the order of tens or hundreds of hertz. Specifically, in these particular tests on understanding the the effects of compositional noise, relevant frequencies are lower than 300 Hz^37^. A range of injection perturbation frequencies from 2 to 250 Hz is investigated, with approximate mass fractions of 4% $\le$ $Y_{\mathrm{i}}$ $\le$ 21% (based on the approximate injection flow rates, which emulate realistic amplitudes of convecting combustor fluctuations^65,66^). The valve duty cycle was varied for each case to try to ensure that the injected pulse width was constant for all cases ($t_p$ = 2 ms). For all test cases, the mean flow was at isothermal conditions and the air mass flow rate was set to $\bar{\dot{m}}$ = 6 g s$^{-1}$, which translates to a nominal mean flow bulk velocity of $\bar{u}$ = 3.7 m/s and mean absolute pressure of $\bar{p}$ = 109 kPa in the test section (prior to the unsteady injection of methane). The acquisition window was limited to 1 second (i.e. 1500 shots or data points since $f_{\mathrm{rep}}$ = 1500 Hz). Table 2 summarizes the test conditions.

**Table 2:** Test cases for unsteady experiments: pulse train injection frequency $f_{\mathrm{i}}$, approximate injected mass fraction of methane $Y_{\mathrm{i}}$, number of pulses during the 1 s acquisition window, valve duty cycle, high speed 2 port 80 W SX10 series valve model, maximum flow rate, and response time (on/off) at 0.25 MPa supply pressure and 50% duty cycle.

Case	Injection				Valve			
	$f_{\mathrm{i}}$ (Hz)	$Y_{\mathrm{i}}$ (%)	No. pulses (–)	Duty cycle (%)	Model (–)	Flow rate (L/min)	On (ms)	Off (ms)
1	2	4	2	0.4	-J	150	0.60	0.75
2	10	4	10	2.0	-J	150	0.60	0.75
3	30	4	30	6.0	-E	100	0.55	0.55
4	60	4	60	12.0	-E	100	0.55	0.55
5	100	8	100	20.0	-E	100	0.55	0.55
6	125	8	125	25.0	-E	100	0.55	0.55
7	187.5	17	187	37.5	-A	50	0.45	0.40
8	250	21	250	50.0	-A	50	0.45	0.40

### Phase-averaged signal

In order to improve the signal-to-noise of the measurements, the counts normalized by the shot-to shot laser intensity fluctuations *C* (see Eq. (4)) from multiple injection pulse trains $N_{t}$ were phase-averaged, starting from an origin $t=0$ at the start of each injection pulse train:

9 $$\begin{aligned} \begin{aligned} \bar{C}^b_{{S}} (t) = \frac{1}{N_{t}} \sum _{i=1}^{N_t} C_{i} (t) \qquad \text {for} \qquad 1 \le i \le {N_{t}}. \end{aligned} \end{aligned}$$

After this step, the background is removed using the procedure described in section “Data processing”. The background-subtracted signal $\hat{C}_{S'}$ is integrated over the range of wavelengths relevant to each mode ($\Delta \nu _1$ to $\Delta \nu _2$ for every shot) to obtain the phase-averaged integrated counts:

10 $$\begin{aligned} \begin{aligned} \hat{C}_{S^{\prime }} (t) = \int _{\Delta \tilde{\nu }_1}^{\Delta \tilde{\nu }_2} \bar{C}^b_{{S'}}(t)\,d\tilde{\nu }. \end{aligned} \end{aligned}$$

**Figure 4:**
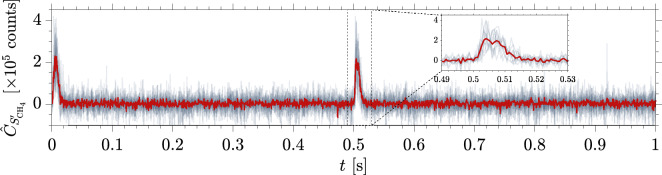
Time-dependent integrated counts $\hat{C}_{S'_{\mathrm{CH_4}}}$ for $f_{\mathrm{i}}$ = 2 Hz injection of methane: time-resolved single-shot () and phase-averaged over $N_t$ = 10 injected pulse trains ().

Figure 4 shows the time-resolved single-shot counts and phase-averaged ($N_t$ = 10 injected pulse trains) integrated counts for the 2 Hz injection of methane (Case 1, as outlined in Table 2).

During the unsteady experiments measuring the simultaneous concentrations of N_2_ and CH$_4$, it was observed that use of the original steady calibration curves led to a total species molar fraction sum that was lower than unity. The deterioration of signal was similar for both methane and nitrogen, and may have been be due to minor deviations of the optics and quartz tube, quartz glass and other etching defects in the mirrors inside the laser. In order to correct for these effects, a mean correction factor of 0.69 was estimated and used as a multiplier to the steady-state calibration curves presented in section “Calibration measurements” by Eqs. (7)–(8). The correction factor was obtained by (a) obtaining the total concentration and thus molar fractions for methane and nitrogen measured from the original steady-state calibration curves, (b) determining the correction factor required to bring all species molar fractions to add up to unity for each unsteady test case (assuming that the signal for both species are equally affected), and (c) using a mean correction factor for all unsteady test cases ($\sigma$ = 0.06). The maximum deviation from unity sum for the molar fractions varied from 0 to 19% for all cases after correction.

### Measurement of unsteady concentration fluctuations

**Figure 5:**
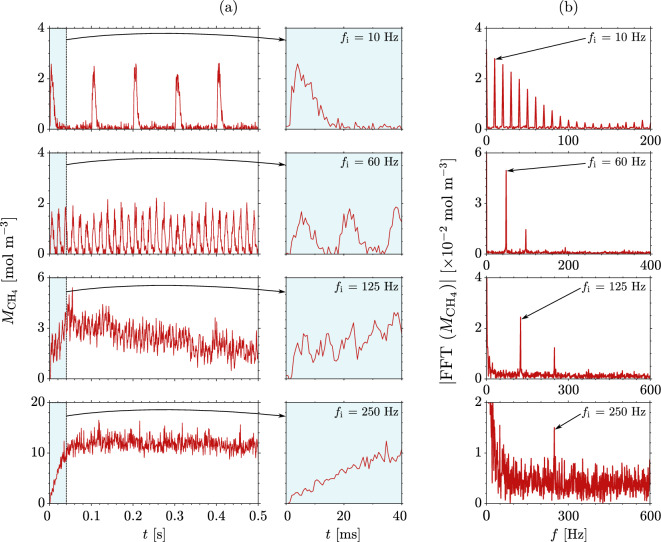
Phase-averaged measurements (${N_t}$ = 10 injected pulse trains) obtained during unsteady co-flow injection of methane at frequency $f_{\mathrm{i}}$ into a mean flow of air at $\bar{\dot{m}}$ = 6 g s$^{-1}$: (**a**) concentration fluctuations ()—half of the full acquisition window (1 s) is shown with the first 40 ms highlighted and zoomed in blue; (**b**) single-side spectrum of the fast Fourier transform of the measured concentration fluctuations for the full acquisition window ().

Figure 5a shows the phase-averaged ($N_t$ = 10 injected pulse trains) Raman measurements of concentration fluctuations during the pulse train injection of methane for a select four of the eight tests. For the cases in which the data acquisition frequency is much higher than the injection frequency, it is possible to resolve the injection fluctuations in time. The details of the injection fluctuations are well resolved, as seen in the time history of concentrations (middle column) for 10 Hz and 60 Hz. Differences in the measured peak amplitude between the 10 Hz and 60 Hz cases (both for $Y_{\mathrm{i}}$ = 4%) may reflect the merging and mixing of injected plumes for the different frequencies. This could be attributed to valve behavior which varies depending on valve model and operating conditions, including duty cycle (see Table 2). For both cases, a Fourier transform of the signals captures traces of the injection frequency (Fig. 5b). For the former, the Fourier transform also resolves and captures several harmonics of the injection frequency. The presence of harmonics arises because of the non-sinusoidal excitation (i.e. square pulses) generated by the injection process. For higher frequencies (125 Hz and 250 Hz), the fluctuations merge via axial mixing, eventually approaching a constant concentration and discerning individual pulse peaks from the baseline of mixed gases becomes more difficult. Nevertheless, the Fourier transform of the signals still captures traces of the injection frequency, even at the highest frequency tested (Fig. 5b).

At the higher frequencies ($f_{\mathrm{i}}$ >125 Hz; third and fourth row), we observe that the mean concentrations rise from zero to a steady state value. This reflects the march to steady-state as the adjusted flow pattern near the nozzle inlet accommodates the additional flow rate of methane and the mixing pattern is well developed. This is reached more quickly for 250 Hz injection (fourth row) due to the higher valve duty cycle (see Table 2), as the short and frequent pulses merge into a nearly constant concentration stream, whereas in the case of 125 Hz, there is an interaction between the pulsed injection and the flow through the nozzle, leading to a longer time for a repeatable pattern to establish itself.

The coefficient of variation of the integrated counts is estimated to be, on average, approximately 28% for nitrogen and 35% for methane. However, it is important to recognize that, since there is a process of phase-averaging, the unsteady injection variance is also a result of actual physical variance due to the turbulent injection of a gas, rather than solely due to calibration or random error.

These experiments demonstrate that spontaneous Raman spectroscopy can be successfully used to acquire the time signatures of injected concentration fluctuations at frequencies of interest for unsteady processes, with the additional advantage of capturing multiple species.

## Conclusions

This investigation demonstrates the application of a high-frequency, low energy visible pulsed laser for gas phase spontaneous Raman spectroscopy to measure and resolve convecting concentration fluctuations at frequencies up to 250 Hz in an isothermal binary gas mixture of methane and air. The original motivation for the work was the study of the indirect noise generated by the acceleration of compositional inhomogeneties and the subsequent need to measure the amplitude of compositional inhomogeneities in a time-resolved manner. This was achieved by using a relatively simple single-camera setup which allowed for the simultaneous multispecies (${\mathrm N}_2$ and ${\mathrm C}{\mathrm H}_4$) measurement on a millisecond time scale, for a repeatable cycle by phase averaging.

Using steady-state experiments, a maximum counts-based SNR of 4.4 for nitrogen and 10.6 for methane was measured, with (coefficients of variation of 22.5% and 9.5%, respectively). The calibration was employed in unsteady experiments at up to 250 Hz, in which it was possible to detect phase-averaged fluctuations. Avenues for further improvement in SNR for future steady-state and unsteady measurements using this measurement technique are possible. The use of an intensifier with higher sensitivity at the relevant scattered wavelengths would improve SNR significantly since the quantum efficiency of the available intensifier was approximately 3% and 2% at the ${\mathrm N}_{2}$ and ${\mathrm C}{\mathrm H}_4$ scattered wavelengths, respectively. Flat windows (rather than the current curved tube) would be preferable to avoid alignment issues and improve collection, as would the use of specialty quartz for low background noise. Larger collection lenses in a relay arrangement into the spectrometer would improve collection efficiency. Further SNR improvement can be achieved for applications at larger pressures. In addition, there is a trade-off in spatial and time resolution and SNR for fixed beam energy delivery. Longer times or larger collection volumes would improve it, at the expense of poorer resolution.

Given that the main limitation of spontaneous Raman measurements is the relatively high energy required, the technique presented here is most appropriate for cases where (i) higher concentrations (of the order of percent at close to ambient conditions) are expected, (ii) millimeter spatial resolution is needed (rather than line-of-sight measurements), (iii) simultaneous measurements of multiple species are desirable, and (iv) pulse-burst lasers are unavailable or cost prohibitive. If one is looking for a more modest, single-camera setup where local measurements on a millisecond time scale are desirable, then this technique can be useful. Example applications include (but are not limited to) gas-phase catalysis, environmental flows, laminar reacting flows, species transport and mixing, and thermoacoustic instabilities.

## Figures and Tables

**Figure 6:**
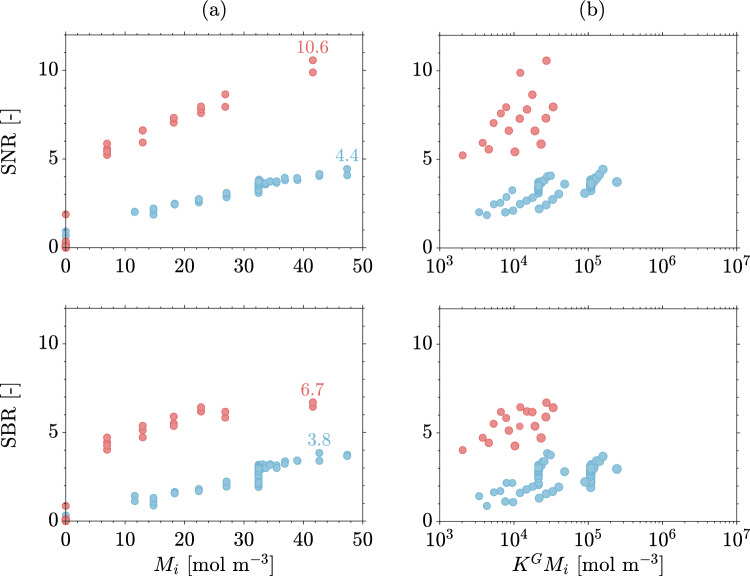
Steady-state experiments: signal-to-noise ratio (SNR) and signal-to-background ratio (SBR) for nitrogen () and methane () in the non-saturated region. Circle sizes are proportional to intensifier gain *G*.

## Data Availability

Data underlying the results presented in this paper are not publicly available at this time but may be obtained from the corresponding author upon reasonable request.

## A Additional details on steady-state calibration results

### A.1 Full calibration curve fits

The processes that govern ICCD imaging systems are well described in literature^67,68^. Indeed, ICCD imaging systems are known to saturate after a specific level of counts^69^. For this system in particular, the camera saturates around 1.45 $\times$ 10$^6$ counts, as shown in Fig. 3a,b. This boundary was calculated using an optimization routine. The non-saturated dataset shows a fit which follows a power-law relationship $\bar{\bar{C}}_{S'_i}$ = $\beta _i (K^{G} M_i)^{\eta }$. Different power-law constants were estimated for a range of boundaries above which counts were no longer considered as part of the fits. Additionally, for accuracy, if for a particular test case the methane data point is saturated, then the nitrogen data point in that test case will also be rejected from the fit (even if it is below the saturation boundary) since, when a particular mode width is saturated, the acquired data was witnessed to move to adjacent wavenumbers, yielding potentially inaccurate results. Two goodness of fit metrics (adjusted $R^2$ and root-mean-square error RMSE) were extracted for each boundary case. The quoted saturation boundary (1.45 $\times$ 10$^6$ counts) was identified by investigating the absolute value of the derivative of the average of both species’ goodness of fit metrics and identifying the first clear maxima which indicates deviation from the power-law fit; the independently extracted index using both goodness of fit metrics were in agreement with each other. Using this procedure, we obtained that $\eta$ = 0.77 is a constant and $\beta _i$ is molecule-specific ($\beta _{\mathrm {CH_4}}$= 165, $\beta _{\mathrm {N_2}}$= 31.4). The power-law constants $\beta$ and $\eta$ are solved for via the minimization of the residual sum of squares using the standard deviation $\sigma$ of the datasets as weighting^70^. Specifically, more sway is given to the data points with least uncertainty via the weighting $\sigma ^{-2}$. For the saturation boundary calculated by the optimization routine, there are 22 methane–air mixture test cases which saturate and an additional 23 cases of pure air flow test cases which also saturate. This means that a total of 122 data points are used for the methane data power-law fit, whereas 99 data points are used for the nitrogen data power-law fit.

### A.2 Signal-to-noise ratio (SNR) and signal-to-background ratio (SBR)

The signal-to-noise ratio (SNR) and the signal-to-background ratio (SBR) for the signals measured as part of the steady-state calibration experiments presented in section “Steady-state experiments” can be defined as:

11a-b $$\begin{aligned} {\mathrm{SNR}} = \dfrac{\bar{\bar{C}}_{S'}}{\sigma _{\bar{\bar{C}}_{S'}}}, \qquad \qquad {\mathrm{SBR}} = \dfrac{\bar{\bar{C}}_{S'}}{\bar{\bar{C}}_{B}}. \end{aligned}$$

Figure 6 shows the range of SNR and SBR measured (cases where the camera saturated are not shown). A maximum SNR of 4.4 and 10.6 was measured for nitrogen and methane, respectively. A maximum SBR of 3.8 and 6.7 was measured for nitrogen and methane, respectively.
